# Platelet count and retinopathy of prematurity, a systematic review and meta-analysis

**DOI:** 10.3389/fped.2024.1413271

**Published:** 2025-01-14

**Authors:** Qian Zeng, Ling Wang, Yurong Li, Yuanyuan Yue, Xin Lv

**Affiliations:** ^1^ Clinical Laboratory, Children’s Hospital Affiliated to Shandong University, Jinan, China; ^2^ Clinical Laboratory, Jinan Children’s Hospital, Jinan, China

**Keywords:** platelet count, retinopathy of premature, ROP, premature, meta-analysis

## Abstract

**Objective:**

This study aims to investigate the relationship between platelet count (PLT) and retinopathy of prematurity (ROP), with the goal of identifying a straightforward screening method for the early detection of ROP.

**Methods:**

A systematic search was conducted in PubMed, EMBASE, Web of Science, Cochrane Library, and ClinicalTrials.gov from January 2005 to 26 September 2023. Subsequently, a subgroup analysis was performed.

**Results:**

This meta-analysis included ten studies involving 1762 neonates, with 747 cases of ROP identified. The pooled analysis indicated a significant decrease in mean platelet count among the ROP group compared to the non-ROP group (mean difference: −18.65, 95% confidence interval: −22.80 to −14.50, *P* < .00001). Subgroup analysis indicated that the heterogeneity in the results can be attributed to factors such as the location of the studies, gestational age (GA), and publication time.

**Conclusion:**

Decreased PLT counts may be associated with the occurrence of ROP. The PLT count should be standardized according to the detection location, detection time and GA.

## 1 Introduction

Retinopathy of prematurity (ROP) is a type of retinal vasoproliferative disease that occurs in premature infants (1). According to the statistics of the World Health Organization, ROP has emerged as one of the leading causes of childhood blindness, contributing to 6%–18% of all cases (2). With the increasing survival of infants with low birth weight (BW) and low gestational age (GA), the incidence of retinopathy of prematurity (ROP) has increased significantly worldwide (3, 4).

Retinal examination is currently the primary method for detecting ROP. However, with the increasing survival rate of premature infants, the workload of ROP screening is also on the rise. There is an urgent need for a rapid and efficient screening method in clinical practice. Recent research has suggested a possible correlation between platelet count and ROP (5–10). When vascular endothelial cells are damaged, platelets will immediately perceive and quickly aggregate to the damaged site to form a stable connection with endothelial cells. Afterwards, platelets undergo deformation, degranulation, and release various key vascular growth factors that are involved in the process of angiogenesis (11). Keşkek et al. (12) found that decreased platelet counts in the first week after birth was a risk factor for the development of ROP and was associated with the progression of the condition to severe ROP in a retrospective study. However, Ozturk et al. (6) analyzed 120 cases of premature infants and reached different conclusions: blood analysis results at gestational age 36 weeks’ showed that the platelet count in the ROP group was higher than that in the non-ROP group (282.74 ± 116.55 vs. 262.05 ± 98.44). The role of PLT count in ROP remains controversial, with scholars continuing to search for their statistical significance and clinical correlation.

We conducted this meta-analysis to clarify the relationship between PLT count and ROP, expanding our understanding of the role of PLT count in ROP and providing new screening methods for ROP.

## 2 Methods

### 2.1 Search strategies

This meta-analysis was performed according the guidelines of Preferred Reporting Items for Systematic Reviews and Meta-analysis (PRISMA) (13). Two reviewers (ZQ and LYR) independently conducted a comprehensive search of the electronic databases, including PubMed, EMBASE, Web of Science, Cochrane Library and ClinicalTrials.gov, to identify all relevant literature published from January 2005 to 26 September 2023. We also did a manual search, using the reference lists of key articles published in English. The following mesh terms and related synonyms were used in the search: (“retinopathy of prematurity” or “Prematurity Retinopathies” or “Prematurity Retinopathy” or “Retrolental Fibroplasia” or “ROP”) and (“platelet” or “thrombocyte” or “thrombocytopenia” or “plt” or “PLT”). Online registration was available at http://www.crd.york.ac.uk/PROSPERO/ (registration number: CRD42023485193).

### 2.2 Selection criteria

Firstly, ZQ and LYR use Endnote to remove duplicate records and screened the titles and abstracts of the remaining articles; then, the potentially relevant reports were assessed based on our inclusion criteria: (1) Study design: original observational studies (case-control, cohort studies, or retrospective studies) or randomized controlled trials evaluating the correlation between ROP and platelet count; (2) Participants: only studies that the diagnosis of ROP was based on International Classification of Retinopathy of Prematurity (ICROP) were included (14). The infants were divided into the case group (ROP) and the control group (non-ROP) based on standard ROP examinations; (3) Outcome variables: platelet count which could be calculated as mean ± standard deviation; (4) Include articles in English only. The exclusion criteria included the following: (1) Articles that were reviews, case reports, letters, comments, abstracts, or nonhuman investigations; (2) Reports that provided insufficient data; (3) Studies for which the original data could not be calculated; (4) Studies conducted on the same population; (5) Studies lacking a control group; (6) Studies with a Newcastle-Ottawa Scale (NOS) score of less than 5.

### 2.3 Data extraction

ZQ and LYR extracted data from the eligible studies included studies using a predesigned data collection form. The following parameters were extracted: primary author, year of publication, study design, country, detection time, sample size and mean ± standard deviation of PLT count for each group, gestational age and birth weight of enrolled infants. Any discrepancies were resolved by discuss with a third investigator. We used formulas to convert the median, quartile, range, 95% confidence interval, etc. in the study into mean and standard deviation (15, 16).

### 2.4 Quality and risk of bias assessment

ZQ and LYR independently evaluated the methodological quality of each study using the Newcastle-Ottawa Scale (NOS) (17). This scale consists of 3 aspects: selection of the study groups (0–4 points), comparability of the groups (0–2 points), and ascertainment of the exposure or outcome (0–3 points). A score of 5 or above was considered as satisfactory quality. Since only observational studies were included, we also used NOS to assess the risk of bias. The final evaluation results indicated that all included 10 studies attained a total NOS score of 6–7 (Supplementary Table 1).

### 2.5 Statistical analysis

This meta-analysis was conducted in the statistical program RevMan 5.1 software, using mean difference and the corresponding 95% CI to calculate the continuous outcomes. The Cochran's Q test and I-square test were used to examine the cohort heterogeneity. The Q statistic was considered significant if *P* < 0.1 and I_2_ above 50% indicated high heterogeneity and random-effect modeling was used for analysis; Otherwise, a fixed model was used. The results were presented as Z values, each corresponding to a *P* value; *P* values less than 0.05 were taken to indicate significant differences. If significant heterogeneity was found between studies, subgroup analysis was performed based on factors (detection time, location, GA and publication year) that could potentially impact the heterogeneity to reduce the sources of heterogeneity.

### 2.6 Sensitivity analysis and publication bias

A sensitivity analysis was performed using the method of removing one study at a time to assess the robustness of the results. Funnel plots were used to evaluate the presence of publication bias.

### 2.7 Certainty of evidence

Two investigators (LW and YYY) independently evaluated the overall credibility of each outcome estimate using the Grading of Recommendations, Assessment, Development and Evaluation (GRADE) Working Group system. This assessment considered study design, directness of evidence, consistency, precision, and potential outcomes related to publication bias.

## 3 Results

### 3.1 Search results

**Figure 1:**
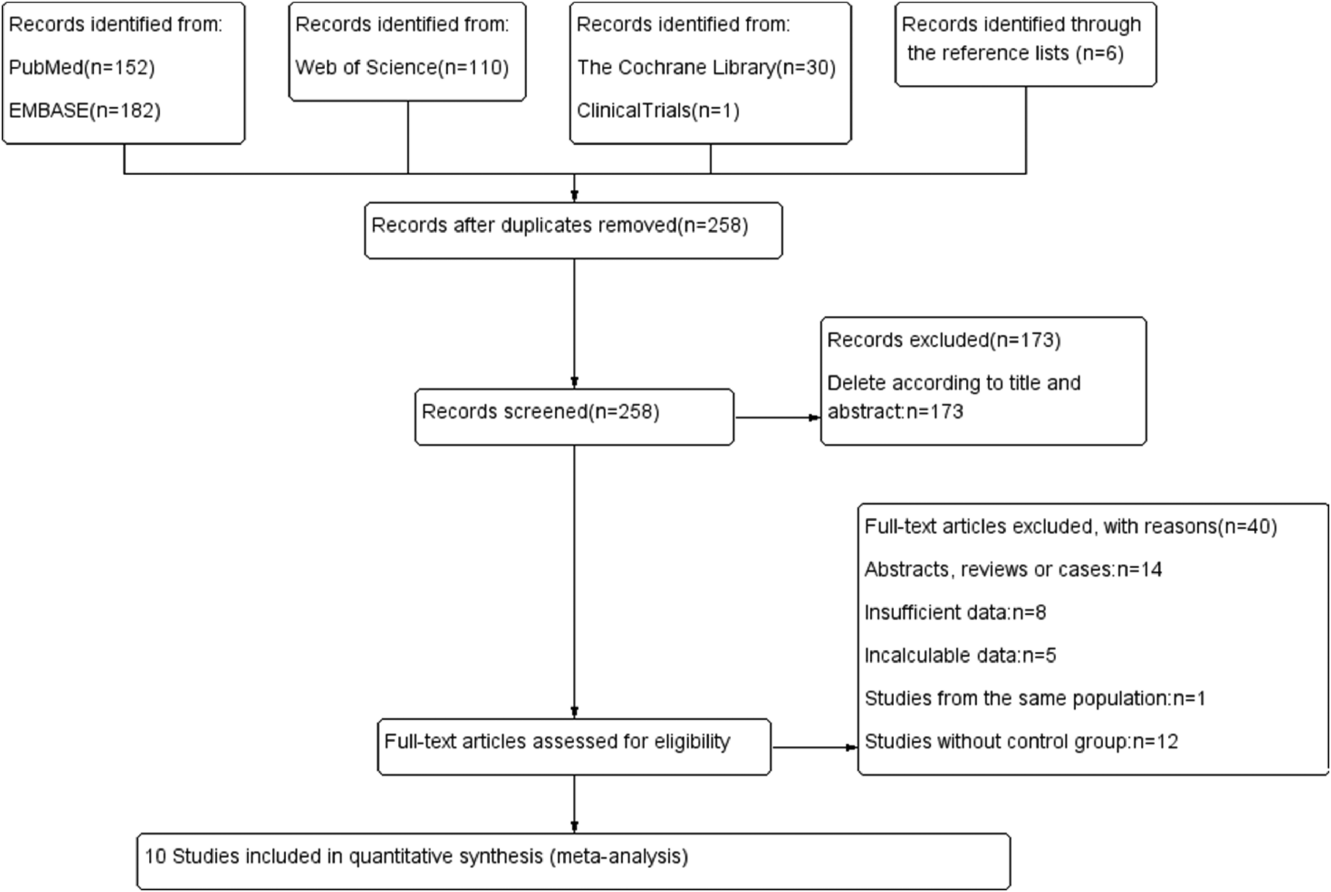
Flow chart.

The flow chart of the literature search and selection process is illustrated in Figure 1.

Initially, 481 potentially relevant studies were identified. After eliminating 258 duplicate articles, 173 studies were excluded based on title and abstract review, leaving 50 publications for further assessment. The reasons for further exclusion included abstracts, reviews, letters, and cases studies (*n* = 14); insufficient data (*n* = 8); incalculable data (*n* = 5); studies from the same population (*n* = 1); and studies without a control group (*n* = 12). Finally, 10 articles (5, 6, 12, 18–24) with 1,762 participants were included in the meta-analysis (Table 1).

**Table 1:** General characteristic of included study.

Author	Year	Study design	Country	Detection time	ROP		Control		Gestational age	Birth weight	NOS
					Total	platelet count (10^9^/L)	Total	platelet count (10^9^/L)			
Ekinci (5)	2023	Retrospective case-control study	Turkey	Postnatal 1st week	80	208 ± 11	51	227 ± 15.1	<36 w	NM	7
				Postnatal 1st month	80	333 ± 19	51	388 ± 24.9	<36 w	NM	
Ünsal (18)	2019	Retrospective case-control study	Turkey	Postnatal 1st month	99	324.89 ± 147.45	43	348.29 ± 168.73	<36 w	<2,670 g	6
Ozturk (6)	2021	Retrospective case-control study	Turkey	Postnatal 24 h	86	192.29 ± 97.78	34	174.97 ± 68.78	<36 w	<2,730 g	6
				Gestational Age 36 w	86	282.74 ± 116.55	34	262.05 ± 98.44	<36 w	<2,730 g	
Keşkek (12)	2020	Retrospective case-control study	Turkey	Postnatal 1st week	47	219.45 ± 66.28	90	280 ± 103	<34 w	NM	6
Parrozzani (19)	2021	Retrospective case-control study	Italy	Postnatal 1.5 h	206	190.83 ± 79.41	357	210.16 ± 72.37	<36 w	<1,985 g	6
Hu (20)	2017	Retrospective case-control study	China	Postnatal 24 h	40	264.53 ± 95.72	40	261.91 ± 80.57	<32 w	<1,900 g	6
Özkaya (21)	2022	Retrospective case-control study	Turkey	Before ROP treatment	80	302.88 ± 130.80	40	310 ± 119.41	<33 w	<2,620 g	6
Okur (22)	2015	Retrospective case-control study	Turkey	Postnatal 24 h	55	195 ± 71	275	221 ± 82	<32 w	NM	6
				Postnatal 1st week	55	210 ± 121	275	258 ± 138	<32 w	NM	
Lubetzky (23)	2,005	Retrospective case-control study	Israel	Postnatal 1 h	23	245.1 ± 67	23	243.9 ± 51.6	<34 w	<2,625 g	7
Lim (24)	2020	Retrospective case-control study	Malaysia	Postnatal 1st week	31	179.8 ± 53.9	62	190.8 ± 48.4	<32 w	<1,500 g	7
				Postnatal 1st month	31	314.9 ± 151	62	399.2 ± 135	<32 w	<1,500 g	

NOS, Newcastle-Ottawa Scale; NM, Not mentioned; w, weeks.

### 3.2 Study characteristics and quality assessments

**Figure 2:**
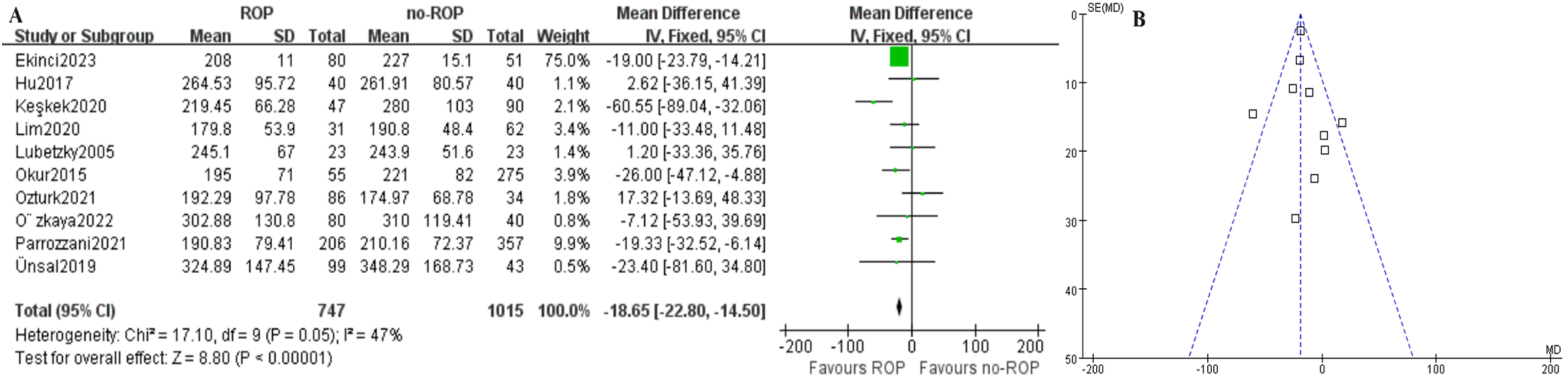
Mean PLT count between case and control group. **(A)** Forest plot. SD, standard deviation; CI, confidence interval; df, degree of freedom; I^2^, extent of inconsistency; Z, overall effect. **(B)** Funnel plot.

The characteristics of the 10 studies included in this analysis are summarized in Table 1. These studies were published between 2005 and 2023 and involved a total of 747 patients and 1,015 controls. Six of the studies were conducted in Turkey, while the remaining four were conducted in other countries. All included studies had a retrospective case-control design and focused on blood platelet counts. When multiple platelet counts were performed on participants, the results from the initial and postpartum months or before ROP treatment were considered. ROP diagnosis was established through fundus examination, with patient classification according to the ICROP (2005) guidelines. Seven studies received NOS scores of 6 indicating moderate quality, while three studies received NOS scores of 7, suggesting high quality. Funnel plot tests for publication bias showed no significant bias (Figure 2B).

### 3.3 The mean PLT count between case and control group

In the ROP group, the mean platelet count (10^9^/L) was found to be significantly lower compared to the non-ROP group (MD: −18.65, 95% CI: −22.80 to −14.50, *P* < .00001), as shown in Figure 2A. Despite this, there was still moderate heterogeneity present (*P* = 0.05, I_2_ = 47%). Subgroup analysis was then conducted.

### 3.4 The mean PLT count at different severity of ROP

**Figure 3:**
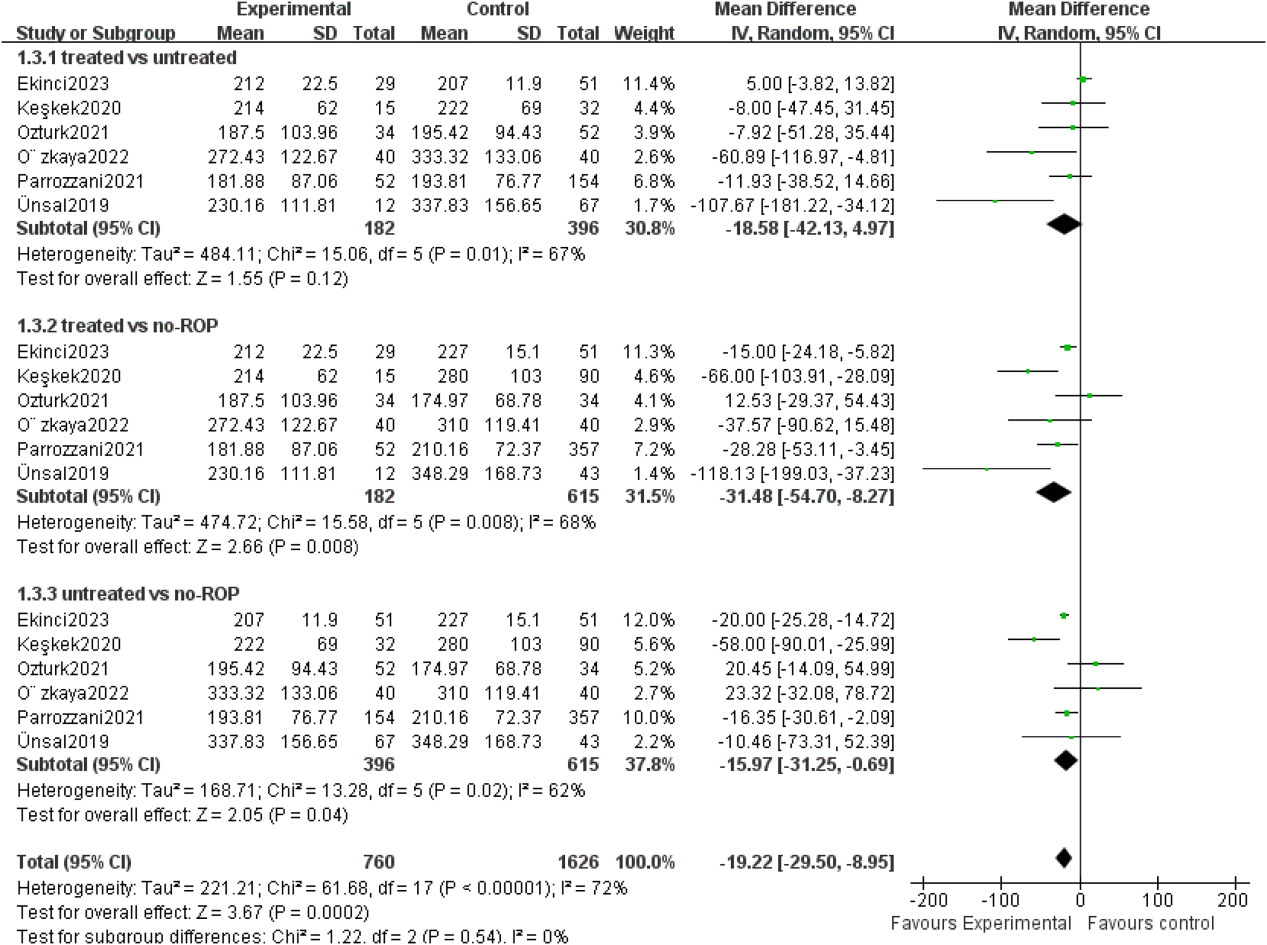
Mean PLT count at different severity of ROP. SD, standard deviation; CI, confidence interval; df, degree of freedom; I^2^, extent of inconsistency; Z, overall effect.

Six studies compared PLT counts among treated ROP, untreated ROP and non-ROP groups (Supplementary Table 2). The platelet count in the treated group was significantly lower than in the non-ROP group, and the count in the untreated ROP group was significantly lower than in the non-ROP group. There was no signiﬁcant association between the treated ROP group and the untreated ROP group (*P* = 0.008, *P* = 0.04, *P* = 0.12) (Figure 3). These findings suggest that the ranking of PLT counts may be treated ROP<untreated ROP<non-ROP.

### 3.5 Subgroup analysis of platelet count

#### 3.5.1 Subgroup by the detection time

**Figure 4:**
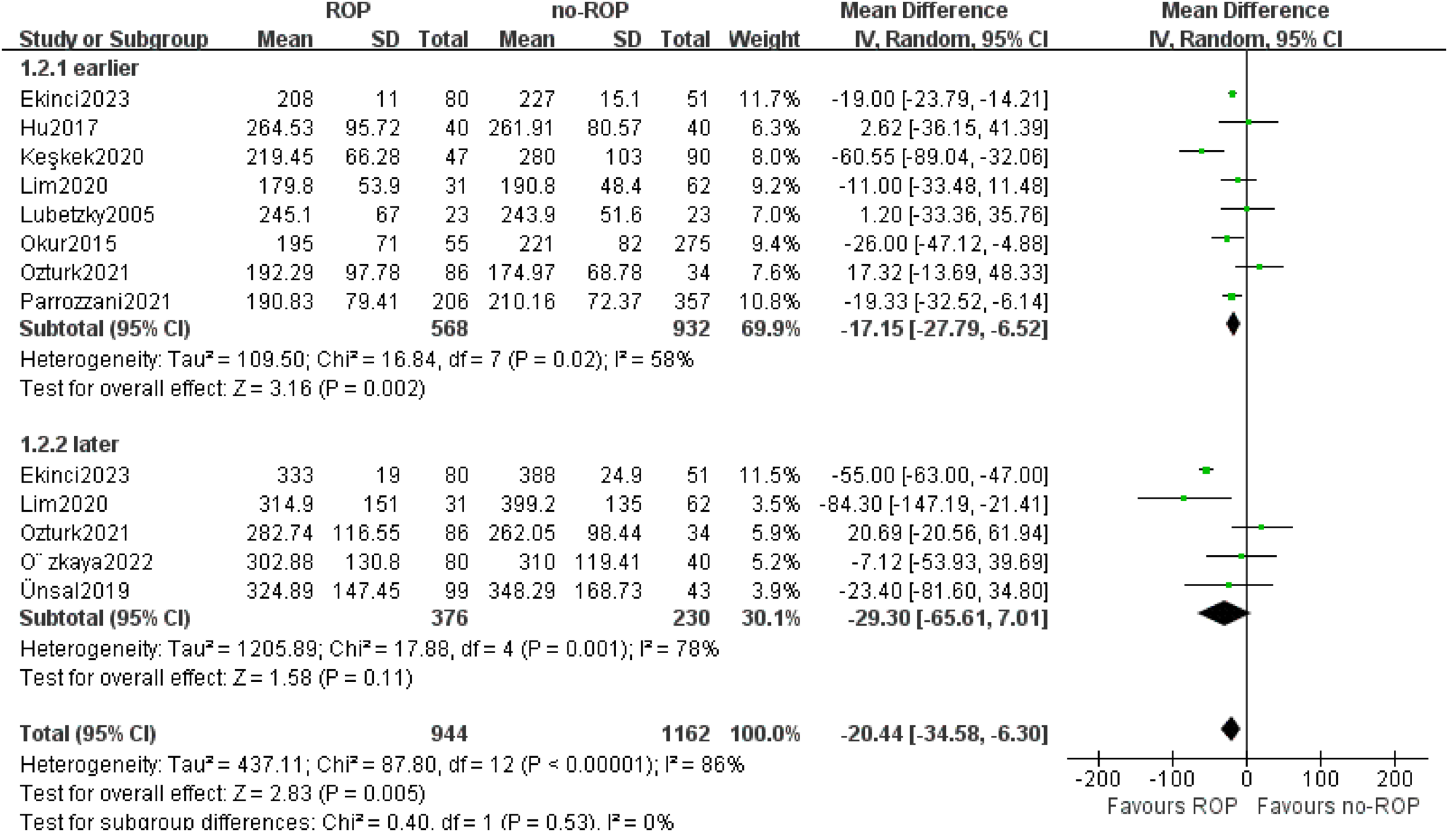
Subgroup by the detection time. SD, standard deviation; CI, confidence interval; df, degree of freedom; I^2^, extent of inconsistency; Z, overall effect.

Out of the 10 studies analyzed, 4 examined platelet (PLT) counts at two different time points. The testing period was categorized into early and late-stage subgroups, defined as within 10 days and 1 month after birth, respectively. In the Okur study, both time points fell within the early stage, resulting in a total of 13 data groups included in the subgroup analysis. The findings indicated a significantly lower platelet count in children with ROP compared to those without ROP in the early-stage subgroup (MD: −17.15, 95% CI: −27.79 to −6.52, *P* = 0.002). While 5 studies were part of the late-stage subgroup, no significant differences were observed (MD: −29.30, 95% CI: −65.61–7.01, *P* = 0.11). Notably, there was still considerable heterogeneity in the data analysis (*P* < 0.00001, I2 = 86%, Figure 4).

#### 3.5.2 Subgroup by the location

**Figure 5:**
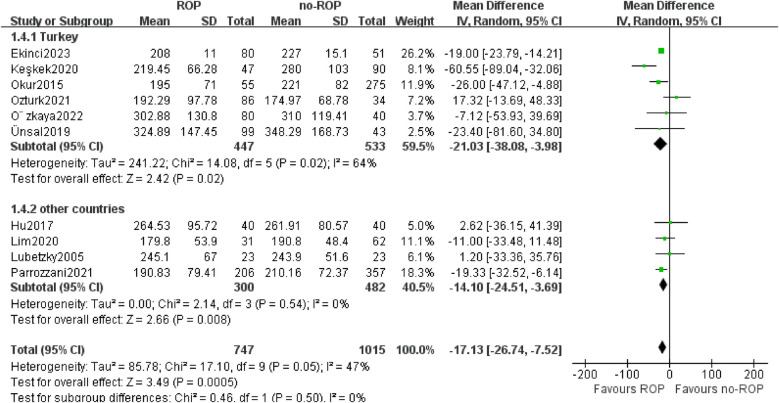
Subgroup by the location.SD, standard deviation; CI, confidence interval; df, degree of freedom; I^2^, extent of inconsistency; Z, overall effect.

Out of the 10 articles analyzed, 6 focused on children from Turkey while 4 involved children from other countries. The analysis revealed a significant decrease in PLT count in ROP children compared to non-ROP controls in the Turkey subgroup (MD: −21.03, 95% CI: −38.08 to −3.98, *P* = 0.02) and the subgroup from other countries (MD: −14.10, 95% CI: −24.51 to −3.69, *P* = 0.008). The heterogeneity disappeared in the subgroup from other countries (*P* = 0.54, I_2_ = 0%, Figure 5).

#### 3.5.3 Subgroup by the gestational age (Ga)

**Figure 6:**
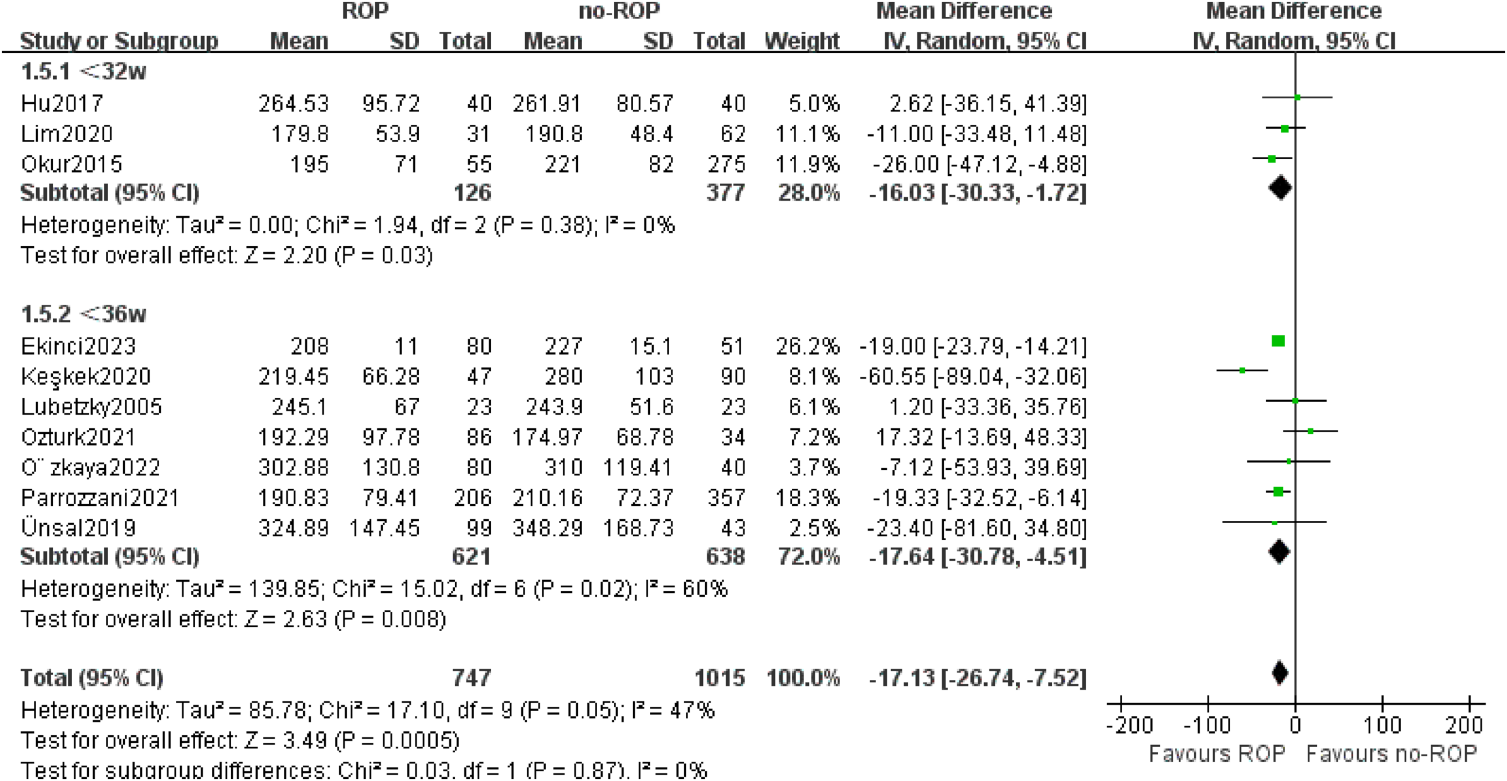
Subgroup by the GA.SD, standard deviation; CI, confidence interval; df, degree of freedom; I^2^, extent of inconsistency; Z, overall effect.

Based on the GA of enrolled children in the study, we separated 10 studies into two subgroups: GA less than 32w and GA between 32–36w. PLT counts were found to be lower in the ROP group compared to the non-ROP group in both subgroups (32w MD: −16.03, 95% CI: −30.00 to −1.72, *P* = 0.03; 32–36w MD: −17.64, 95% CI: −30.78 to −4.51, *P* = 0.008). The heterogeneity was not present in the GA less than 32w subgroup (*P* = 0.38, I_2_ = 0%, Figure 6).

#### 3.5.4 Subgroup by the year of publication

**Figure 7:**
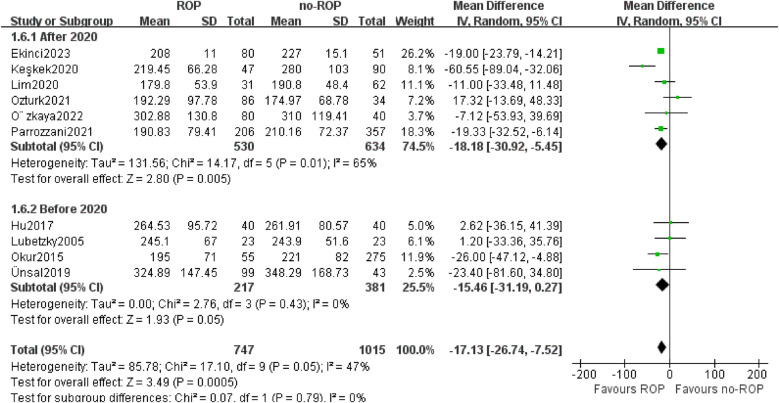
Subgroup by the publication year.SD, standard deviation; CI, confidence interval; df, degree of freedom; I^2^, extent of inconsistency; Z, overall effect.

Out of the 10 studies included, 4 were published before 2020, while the other 6 were published after 2020. Therefore, we categorized the studies into two subgroups based on the 2020 boundaries. The findings from both subgroups indicated a significant decrease in platelet count among children in the ROP group compared to those in the non-ROP group (after 2020 MD: −18.18, 95% CI: −30.92 to −5.45, *P* = 0.005; before 2020 MD: −15.46, 95% CI: −31.19–0.27, *P* = 0.05). Furthermore, the heterogeneity in the before 2020 subgroup was no longer present (*P* = 0.43, I_2_ = 0%, Figure 7).

### 3.6 Grading

All studies included in the meta-analysis were observational; therefore, the baseline grade of overall evidence was deemed low according to the GRADE manual. Nevertheless, based on the positive effect indicators, the evidence level of several studies was classified as moderate. The specific evaluation results are detailed in the supplementary material.

## 4 Discussion

This meta-analysis is the first to investigate the relationship between platelet count and retinopathy of prematurity (ROP). By integrating and analyzing relevant studies, we were able to identify a significant relationship between the two variables. All included articles met high quality standards with complete and reliable data (NOS>5), making them suitable for meta-analysis. The pooled results indicated a significantly lower platelet count in the ROP group compared to the non-ROP group (Figure 2A), suggesting that a low platelet count may be a risk factor for ROP patients. However, moderate heterogeneity was observed in the results. Subgroup analysis was then conducted to explore potential sources of heterogeneity, revealing that patient location, gestational age, and publication time could be contributing factors to the observed variation.

The pathological changes of ROP primarily involve two stages: the first stage includes the impairment of retinal vascular development in premature infants’ post-birth and the occlusion of immature retinal capillaries due to exposure to high oxygen partial pressure. The second stage is characterized by an increased metabolic demand for retinal maturation, leading to elevated levels of vascular endothelial growth factor (VEGF) that stimulate abnormal neovascularization, ultimately resulting in retinal traction, detachment, and potential blindness (25, 26). Previous research has highlighted the crucial role of platelets in these stages (27). Platelets respond promptly to endothelial cell damage, aggregating at the site of injury and releasing key vascular growth factors to promote angiogenesis.

Therefore, this study conducted subgroup analysis on different detection times and gestational age to determine which time point is a more reliable indicator for comparisons. The findings revealed that platelet counts were significantly lower in ROP children compared to non-ROP children in the early-stage subgroup, with no significant differences in the later-stage subgroup. Additionally, both gestational age subgroups showed lower platelet counts in the ROP group compared to the non-ROP group. These results indicate a notable decrease in platelet count in ROP children, particularly evident when tested within 10 days after birth.

Two common heterogeneity factors, location and publication time, were analyzed, revealing them as sources of heterogeneity. Subgroup analysis of other countries and publication time before 2020 showed the disappearance of heterogeneity. Despite some variations, the results consistently indicated that platelet count in ROP patients was significantly lower than in non-ROP patients, irrespective of country or publication year. We believed that the observed heterogeneity following 2020 could be attributed to the relatively small number of studies conducted, as well as various other factors influencing ROP in each study, including low birth weight and gestational age.

Several studies have highlighted the association between platelet count and the development and the severity of ROP. The findings suggested that the ranking of platelet count may be as follows: ROP treated <untreated ROP<non-ROP. This was consistent with the results of many previous studies (28–30).

There are several limitations in our study. Firstly, the results are based on observational studies as there are no relevant RCT studies available. This study design does not allow for a true assessment of causality between the two variables due to potential bias and confounding factors. Publication bias was inevitable, increasing the likelihood of missing negative results from unpublished studies. Additionally, non-English studies were excluded, and no contact was made with authors or experts.

## 5 Conclusion

Decreased PLT counts may be associated with the occurrence of ROP and are correlated with the severity of ROP. It is recommended to standardize the PLT count by considering the location, detection time, and gestational age, as well as establishing reference intervals to differentiate between ROP and non-ROP cases. Further research is required to validate our findings and enhance the conclusions of this study.
