# Supplementary material for: Platelet count and retinopathy of prematurity, a systematic review and meta-analysis
Source: Front Pediatr. 2025 Jan 14;12:1413271. doi: 10.3389/fped.2024.1413271 (PMC11772428; doi:10.3389/fped.2024.1413271)
Supplement: Supplementary file 1 [file Datasheet1.docx]

Supplementary table 1: Evaluation result of the Newcastle-Ottawa Scale.

| **Study** | **Selection** | | | | **Comparability** | **Exposure** | | | **Scores** |
| --- | --- | --- | --- | --- | --- | --- | --- | --- | --- |
|  | Adequate definition of cases | Representativ-eness of cases | Selection of controls | Definition of controls | Control for important factor | Ascertainment of exposure | Same method of ascertainment for cases and controls | Nonresponse rate |  |
| Ekinci | ☆ | ☆ |  | ☆ | ☆ | ☆ | ☆ | ☆ | 7 |
| Ünsal | ☆ | ☆ |  | ☆ |  | ☆ | ☆ | ☆ | 6 |
| Ozturk | ☆ | ☆ |  | ☆ |  | ☆ | ☆ | ☆ | 6 |
| Keşkek | ☆ | ☆ |  | ☆ |  | ☆ | ☆ | ☆ | 6 |
| Parrozzani | ☆ | ☆ |  | ☆ |  | ☆ | ☆ | ☆ | 6 |
| Hu | ☆ | ☆ |  | ☆ |  | ☆ | ☆ | ☆ | 6 |
| O¨ zkaya | ☆ | ☆ |  | ☆ |  | ☆ | ☆ | ☆ | 6 |
| Okur | ☆ | ☆ |  | ☆ |  | ☆ | ☆ | ☆ | 6 |
| Lubetzky | ☆ | ☆ |  | ☆ | ☆ | ☆ | ☆ | ☆ | 7 |
| Lim | ☆ | ☆ |  | ☆ | ☆ | ☆ | ☆ | ☆ | 7 |

Supplementary table 2: General characteristic of different levels of severity of infants.

| **Author** | **Year** | **Detection Time** |  | **Group** | | |  |  |
| --- | --- | --- | --- | --- | --- | --- | --- | --- |
|  |  |  | **Total** | **treated ROP** | **Total** | **untreated ROP** | **Total** | **non-ROP** |
| Ekinci [5] | 2023 | Postnatal 1st week | 29 | 212±22.5 | 51 | 207±11.9 | 51 | 227±15.1 |
| Ünsal [18] | 2019 | Postnatal 1st month | 12 | 230.16±111.81 | 67 | 337.83±156.65 | 43 | 348.29±168.73 |
| Ozturk [6] | 2021 | Postnatal 24h | 34 | 187.50±103.96 | 52 | 195.42±94.43 | 34 | 174.97±68.78 |
| Keşkek [12] | 2020 | Postnatal 1st week | 15 | 214±62 | 32 | 222±69 | 90 | 280±103 |
| Parrozzani [19] | 2021 | Postnatal 1.5h | 52 | 181.88±87.06 | 154 | 193.81±76.77 | 357 | 210.16±72.37 |
| O¨ zkaya [21] | 2022 | Before ROP treatment | 40 | 272.43±122.67 | 40 | 333.32±133.06 | 40 | 310±119.41 |


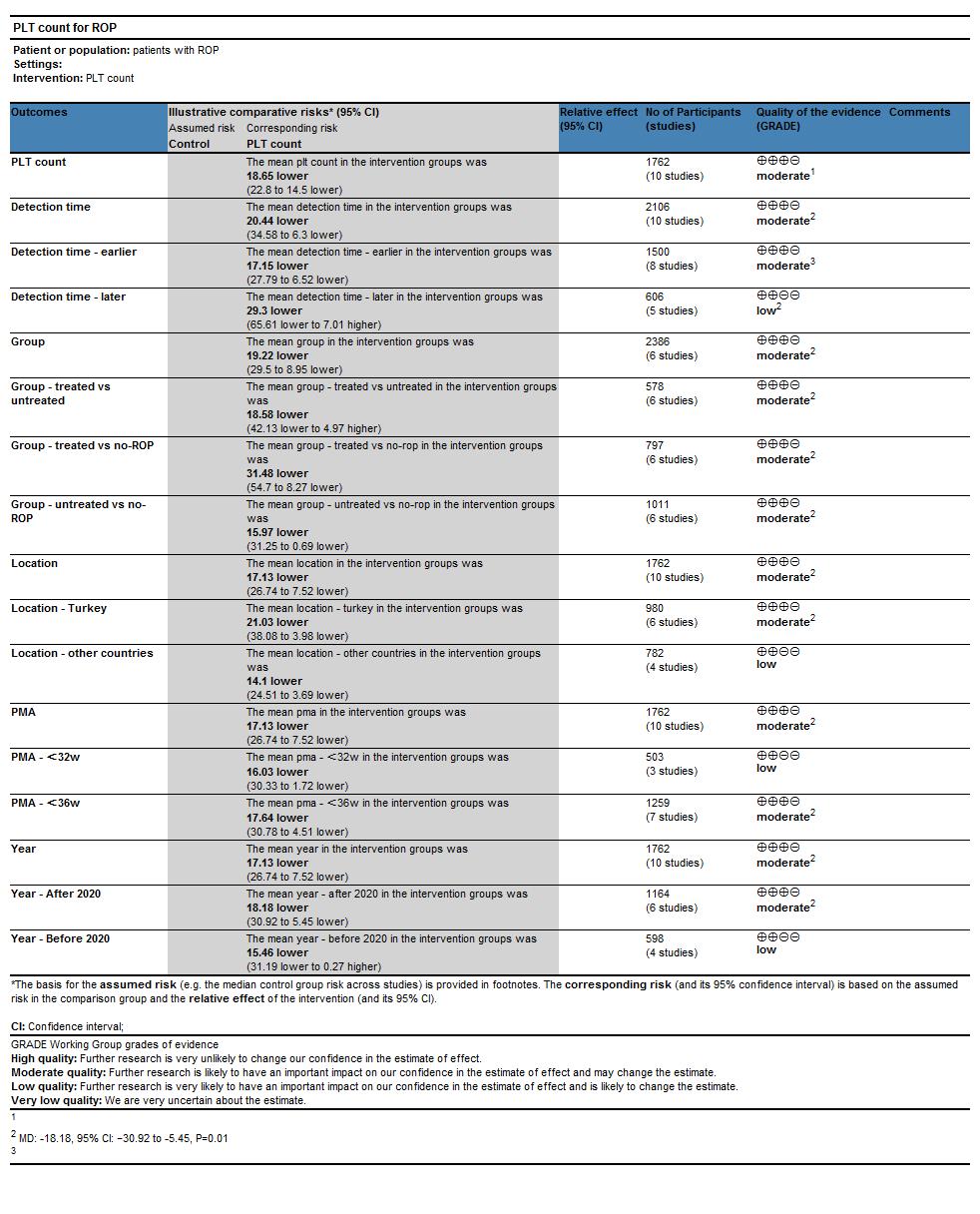


Supplementary figure, GRADE grading.
